# Somatic mutation analysis of *MYH11 *in breast and prostate cancer

**DOI:** 10.1186/1471-2407-8-263

**Published:** 2008-09-17

**Authors:** Pia Alhopuro, Auli Karhu, Robert Winqvist, Kati Waltering, Tapio Visakorpi, Lauri A Aaltonen

**Affiliations:** 1 Department of Medical Genetics and Translational Genome-Scale Biology Research Program, University of Helsinki, Helsinki, Finland; 2 Department of Clinical Genetics, Helsinki University Central Hospital, Helsinki, Finland; 3 Laboratory of Cancer Genetics, Oulu University Hospital and University of Oulu/Biocenter Oulu, Oulu, Finland; 4 Institute of Medical Technology and Tampere University Hospital, University of Tampere, Tampere, Finland

## Abstract

**Background:**

*MYH11 *(also known as *SMMHC*) encodes the smooth-muscle myosin heavy chain, which has a key role in smooth muscle contraction. Inversion at the *MYH11 *locus is one of the most frequent chromosomal aberrations found in acute myeloid leukemia. We have previously shown that *MYH11 *mutations occur in human colorectal cancer, and may also be associated with Peutz-Jeghers syndrome. The mutations found in human intestinal neoplasia result in unregulated proteins with constitutive motor activity, similar to the mutant *myh11 *underlying the zebrafish *meltdown *phenotype characterized by disrupted intestinal architecture. Recently, *MYH1 *and *MYH9 *have been identified as candidate breast cancer genes in a systematic analysis of the breast cancer genome.

**Methods:**

The aim of this study was to investigate the role of somatic *MYH11 *mutations in two common tumor types; breast and prostate cancers. A total of 155 breast cancer and 71 prostate cancer samples were analyzed for those regions in *MYH11 *(altogether 8 exons out of 42 coding exons) that harboured mutations in colorectal cancer in our previous study.

**Results:**

In breast cancer samples only germline alterations were observed. One prostate cancer sample harbored a frameshift mutation c.5798delC, which we have previously shown to result in a protein with unregulated motor activity.

**Conclusion:**

Little evidence for a role of somatic *MYH11 *mutations in the formation of breast or prostate cancers was obtained in this study.

## Background

MYH11 (also known as SMMHC) encodes the smooth-muscle myosin heavy chain and belongs to the family of conventional myosins. The well-characterized biological function of myosins is their ability to use the energy of ATP hydrolysis to move actin filaments and produce muscle force. Recently, myosins have been implicated in a variety of other intra-cellular functions, including cell migration, adhesion, control of cell shape, and membrane traffic [1], as well as in cell-signaling pathways such as interaction with Rho [2] and the pro-apoptotic protein Bmf (BCL2-modifying factor) [3]. In yeast, myosin 5 has been shown to orientate the mitotic spindle making it pivotal in establishment of cell polarity [4]. Furthermore, several recent publications have shown the significance of nuclear actin and myosin I in transcription [1,5].

Inversion at the *MYH11 *locus inv(16)(p13q22) is one of the most frequent chromosomal translocations found in acute myeloid leukemia (AML) and accounts for approximately 8% of all AML cases, especially those of the M4Eo subtype [6]. The inversion results in fusion of the first four to five exons of core binding factor β (*CBFβ*) and the C-terminal tailpiece of the *MYH11 *gene and formation of an oncogenic chimeric protein [6].

Recently, *myh11 *was identified as the predisposing gene for the zebrafish recessive lethal *meltdown *phenotype. The *meltdown *mutants develop cystic expansions of the posterior intestine and expanded connective tissue reminiscent of human juvenile polyposis and Peutz-Jeghers syndrome polyps. The mutation observed in the germline of these fish is located in a highly conserved area of the gene and it leads to constitutive activation of the ATPase function, resulting in disruption of the smooth muscle adjacent to posterior intestine [7].

We have previously identified *MYH11 *as a driver gene in human colorectal cancer [8]. We identified a somatic frameshift mutation (c.5798delC) in the *MYH11 *gene in 55% of colorectal cancers (CRCs) with microsatellite instability. *MYH11 *mutations were also found in one microsatellite stable CRC (altogether 30 MSS CRCs analyzed) and in the germline of one patient with Peutz-Jeghers syndrome. Functional assays demonstrated that the mutations (R500L, K1044N and c.5798del/insC) identified in *MYH11 *in colorectal neoplasia resulted in unregulated proteins with actin-activated motor activity, similar to the mutant *myh11 *underlying the zebrafish *meltdown *phenotype [7]. We suggested that MYH11 could play a role in tumor formation by disturbing stem cell differentiation process or through effects on cellular energy balance.

The aim of the current work was to investigate the possible role of somatic *MYH11 *mutations in two other common cancer types, breast and prostate cancer.

## Methods

### Breast cancer samples

A total of 155 breast cancer DNA samples were available for the study. The samples were derived from patients unselected for a possible family history of cancer and diagnosed with mammary gland adenocarcinoma between 1988 and 1994 at the Oulu University Hospital. The individuals had a mean age at diagnosis of 55 years (range 29–89 years) [9]. Tumor samples were evaluated by a pathologist prior to DNA extraction and contained at minimum 30% of tumor cells. A total of 34 samples (21.9%) had a tumor content < 50% (approximately 40% of tumor cells). On average the tumor cell content was 50–70%, which should enable detection of somatic mutations by sequencing. Patients gave their informed consent, and approval to perform the study was obtained from the Ethical Board of the Northern Ostrobothnia Health Care District (11/2000, 88/2000) and the Finnish Ministry of Social Affairs and Health (Dnr 46/07/98).

### Prostate cancer samples

Genome Phi-amplified (GenomiPhi Amplification kit, Amersham, GE Healthcare, UK) unselected prostate tumour samples (n = 71) from the Tampere region were analysed. Of these 71 samples, 30 were prostatectomy specimens from previously untreated patients. 14 hormone-refractory specimens were obtained from transurethral resection. These patients had been treated by androgen withdrawal, on average, for 38 months (range: 15–68 months). In addition, 6 benign prostate hyperplasia specimens, 16 prostate cancer xenografts (LuCaP 23.1, 23.8, 35, 35V, 49, 58, 69, 70, 73, 77, 78, 81, 92.1, 96, 105, 115, kindly provided by Dr. Robert Vessella, University of Washington, Seattle, WA, USA), and 5 prostate cancer cell lines (DU145, LAPC4, LNCaP, PC-3 and 22Rv1) were available. DU145, LNCaP, PC-3 and 22Rv1 were obtained from the American Type Culture Collection (ATCC, Manassas, VA, USA), and LAPC4 was kindly provided by Dr. Charles Sawyers (MSKCC, New York, NY, USA). Prior to DNA extraction the samples were evaluated by a pathologist to confirm sufficient content of malignant tissue. All tumor samples contained >60% of malignant cells. The use of clinical tumor material has been approved by the Ethical Committee of Tampere University Hospital. All patients participating in this study gave informed consent.

### PCR and sequencing

*MYH11 *(NM_002474 and NM_022844) exons 9, 12, 24, 27, 28, 29, 38 and 40 (isoform SM2) were analyzed using PCR and genomic sequencing. The first coding exon was termed exon 1. *MYH11 *encodes almost 2000 amino acids (42 exons), and targeted mutation analysis was performed focusing only on the regions that were important in colorectal tumor formation. The mutations which were previously functionally examined were located in exons 12 (R500L), 24 (K1044N) and 40 (SM2, c.5798del/insC) [8].

Sequencing primers have been described previously [8]. PCRs were performed in 25 μl volume containing 20 ng of genomic DNA, 1× PCR buffer (Applied Biosystems, AB, Foster City, California), 200 μmol/l each dNTP (Finnzymes, Espoo, Finland), 0.25 μmol/l both primers, and 1.25 units AmpliTaq Gold DNA polymerase. Samples were purified using ExoSAP-IT PCR purification kit (USB Corporation, Cleveland, Ohio), and sequenced using AB 3730 BD3.1 sequencing chemistry and 5.1 sequencing analysis software (Applied Biosystems, Branchburg, NJ). Genomic (un-amplified) DNA was available from the prostate samples for further analyses.

## Results

Most of the studied *MYH11 *exons were successfully analyzed in 80–100% of samples, with a few exceptions. In prostate cancers, exon 9 was successfully analyzed only in 33% of samples due to technical problems, and in breast cancers results from exon 24 sequencing were derived from 73% of cases. As somatic mutations may be missed due to normal tissue contamination, only sequence traces with no background signal were scored successful to enable detection of even small mutation peaks.

Analysis of 155 breast cancer samples for selected *MYH11 *exons revealed no somatic alterations. A heterozygous missense alteration A1072T was detected in one patient with breast cancer, which to our knowledge has not been reported in previous studies (Table 1). This alteration was detected also in the respective germline sample. The significance of A1072T was not evaluated further and remains unknown, although it is likely a rare polymorphism. A missense change D1066N was detected in two breast cancer samples and in the corresponding normal tissue samples in heterozygosity. This variant was observed also in our previous work in the germline DNA of Finnish patients with colorectal cancer and is presumably a polymorphism [8].

**Table 1:** Alterations detected in 155 breast carcinomas and 71 prostate carcinomas (50 primary tumors, 16 xenografts).

**Alteration**	**No of samples**	**Type of alteration (Reference)**
D1066N**	2 Breast ca	Germline polymorphism [8]
A1072T**	1 Breast ca	Germline polymorpism (this study)
A1234T*	28 Prostate ca	Germline polymorphism (Ensembl)
	61 Breast ca	
K1247K**	1 Prostate ca	Unknown (this study)
V1289A*	3 Prostate ca	Germline polymorphism (Ensembl)
	6 Breast ca	
L1323L *	1 Prostate ca	Germline polymorphism (Ensembl)
	10 Breast ca	
A1839A**	7 Breast ca	Germline polymorphism [8]
	1 Prostate ca	
c.5798delC	1 Prostate ca	Candidate somatic mutation

* Reported polymorphism

** Not repoted in Ensembl Genome Browser

A heterozygous alteration K1247K was found in one prostate cancer cell line (Table 1). Unfortunately, no corresponding normal tissue was available and this silent change was not studied further. One prostate cancer sample harbored a frameshift mutation c.5798delC (Table 1) in a microsatellite tract of 8 cytosine units, which we have previously shown to occur frequently in MSI CRC and to result in a protein with unregulated actin-activated motor activity. The sample was re-analyzed using genomic (un-amplified) DNA to confirm the result. Somatic origin of the frameshift mutation or microsatellite-status of this prostate specimen could not be determined due to lack of normal tissue sample. However, in a previous comparative genomic hybridization study [10] the sample showed little signs of chromosomal instability, which could be suggestive of MSI. The sample (LuCaP73) harboring the candidate somatic *MYH11 *frameshift mutation was a xenograft specimen originating from a hormone-refractory prostate cancer. No other somatic changes were detected in prostate cancer samples.

## Discussion

Breast and prostate carcinomas are among the most common cancers in Western countries. The efforts aiming to identify cancer-specific somatic mutations in these cancer types may ideally provide clues to understanding the cellular processes underlying tumor development and lead to diagnostic and therapeutic advances.

Myosins have a well-characterized biological function in the use the energy of ATP hydrolysis to move actin filaments and produce muscle force, but are implicated also in a variety of other cellular functions, many of which are relevant for cancer formation [1]. We have previously shown that somatic mutations in *MYH11*, which result in unregulated proteins with constitutive activity, contribute to CRC formation. Recently, *MYH1 *encoding skeletal muscle myosin heavy polypeptide 1 and *MYH9 *encoding non-muscle myosin heavy chain type A, were identified as candidate breast cancer genes in systematic analyses of the breast cancer genome [10-12]. Here we have extended the analysis of the role of myosins in tumor development and analyzed sizable sets of breast and prostate cancers for somatic mutations in the key regions of the *MYH11 *gene (altogether 8 out of 42 coding exons, covering approximately 18% of the coding region).

We analyzed breast and prostate cancer samples for those *MYH11 *regions that harbored somatic mutations in CRCs. Especially exons 12, 24 and 40 (SM2) were of interest, as we have demonstrated by functional analysis that mutations in these areas result in molecules lacking phosphorylation-dependent regulation. Based on current structural models of myosin regulation, most mutations in the *MYH11 *head or in the rod domain would not be expected to abolish regulation [13]. Therefore it is possible that mutations only in certain regions of *MYH11 *contribute to tumor formation. The strategy was to analyze the regions with the greatest potential for discovery of mutations in a large set of non-CRC tumors.

In this study, no somatic *MYH11 *mutations were found in breast cancer samples. In prostate cancers, an ATPase activating candidate somatic *MYH11 *mutation (c.5798delC) was identified in one xenograft sample only. This mutation may have occurred by chance and therefore more evidence is needed to establish whether *MYH11 *has a role in prostate cancer development. However, c.5798delC seems to occur infrequently in prostate cancers. The negative results are unlikely to be due to normal tissue contamination, as the tumor cell content was typically >50%. Certain somatic mutations, undetectable by sequencing, may have been missed in this study. Based on this work, we cannot exclude the occurrence of mutations in other regions of *MYH11 *and further studies are needed. It would be of interest to study smaller sets of breast and prostate tumors for the entire coding region of *MYH11*. Nevertheless, the study suggests that somatic *MYH11 *mutations are not frequent in breast and prostate cancer.

## Conclusion

In conclusion, somatic mutations in the regions of *MYH11 *that are important for colorectal tumor formation are not frequent in breast or prostate cancers.

## Abbreviations

AML: acute myeloid leukemia; ATCC: American Type Culture Collection; ATP: adenosine triphosphate; BMF: BCL2-modifying factor; CBFβ: core binding factor β; CRC: colorectal cancer; MSI: microsatellite instability; MSS: microsatellite stable; MYH1: skeletal myosin heavy chain, adult 1; MYH9: non-muscle myosin heavy chain, type A; MYH11: smooth-muscle myosin heavy chain; PCR: polymerase chain reaction; SMMHC: smooth-muscle myosin heavy chain.

## Competing interests

The authors declare that they have no competing interests.

## Authors' contributions

PA participated in study design, performed molecular genetic analyses, analyzed the data and wrote the manuscript.

AK participated in study design and the critical revision of the manuscript.

RW, KW, and TV provided specimens and clinical data and participated in the critical revision of the manuscript.

LAA designed the study and participated in manuscript preparation.

All authors read and approved the final manuscript.

## Pre-publication history

The pre-publication history for this paper can be accessed here:


